# IMUP and GPRC5A: two newly identified risk score indicators in pancreatic ductal adenocarcinoma

**DOI:** 10.1186/s12935-021-02324-w

**Published:** 2021-11-24

**Authors:** Rong Wei, Guoye Qi, Zixin Zeng, Ningning Shen, Ziyue Wang, Honghong Shen, Lifang Gao, Chen Song, Wenxia Ma, Chen Wang

**Affiliations:** 1 Department of Pathology, The Second Hospital of ShanXi Medical University, No.382 WuYi Road, Tai Yuan, 030000 ShanXi China; 2 Department of Pathology, The Basic Medical College of ShanXi Medical University, Tai Yuan, ShanXi China

**Keywords:** Pancreatic cancer, IMUP gene, GPRC5A gene, Bioinformatic analysis, GEO database, Independent survival indicator, Molecular pathology

## Abstract

**Background:**

Pancreatic cancer has been a threateningly lethal malignant tumor worldwide. Despite the promising survival improvement in other cancer types attributing to the fast development of molecular precise medicine, the current treatment situation of pancreatic cancer is still woefully challenging since its limited response to neither traditional radiotherapy and chemotherapy nor emerging immunotherapy. The study is to explore potential responsible genes during the development of pancreatic cancer, thus identifying promising gene indicators and probable drug targets.

**Methods:**

Different bioinformatic analysis were used to interpret the genetic events in pancreatic cancer development. Firstly, based on multiple cDNA microarray profiles from Gene Expression Omnibus (GEO) database, the genes with differently mRNA expression in cancer comparing to normal pancreatic tissues were identified, followed by being grouped based on the difference level. Then, GO and KEGG were performed to separately interpret the multiple groups of genes, and further Kaplan–Meier survival and Cox Regression analysis assisted us to scale down the candidate genes and select the potential key genes. Further, the basic physicochemical properties, the association with immune cells infiltration, mutation or other types variations besides expression gap in pancreatic cancer comparing to normal tissues of the selected key genes were analyzed. Moreover, the aberrant changed expression of key genes was validated by immunohistochemistry (IHC) experiment using local hospital tissue microarray samples and the clinical significance was explored based on TCGA clinical data.

**Results:**

Firstly, a total of 22,491 genes were identified to express differently in cancer comparing to normal pancreatic tissues based on 5 cDNA expression profiles, and the difference of 487/22491 genes was over eightfold, and 55/487 genes were shared in multi profiles. Moreover, after genes interpretation which showed the > eightfold genes were mainly related to extracellular matrix structural constituent regulation, Kaplan–Meier survival and Cox-regression analysis were performed continually, and the result indicated that of the 55 extracellular locating genes, GPRC5A and IMUP were the only two independent prognostic indicators of pancreatic cancer. Further, detailed information of IMUP and GPRC5A were analyzed including their physicochemical properties, their expression and variation ratio and their association with immune cells infiltration in cancer, as well as the probable signaling pathways of genes regulation on pancreatic cancer development. Lastly, local IHC experiment performed on PAAD tissue array which was produced with 62 local hospital patients samples confirmed that GPRC5A and IMUP were abnormally up-regulated in pancreatic cancer, which directly associated with worse patients both overall (OS) and recurrence free survival (RFS).

**Conclusions:**

Using multiple bioinformatic analysis as well as local hospital samples validation, we revealed that GPRC5A and IMUP expression were abnormally up-regulated in pancreatic cancer which associated statistical significantly with patients survival, and the genes’ biological features and clinical significance were also explored. However, more detailed experiments and clinical trials are obligatory to support their further potential drug-target role in clinical medical treatment.

**Supplementary Information:**

The online version contains supplementary material available at 10.1186/s12935-021-02324-w.

## Background

Pancreatic cancer has been a common and most importantly lethal malignancy worldwide. The overall 5-year survival rate was 7 ~ 9% and 1-year rate less than 20% [1]. Despite the improvements in clinical treatment strategies for many other cancer types, the treatment methods and survival rate of pancreatic cancer remained steady for the past few decades [2]. Pancreatic cancer has been predicted to be able to surpass breast cancer, colorectal cancer and prostate cancer, becoming the second top cause of cancer related deaths, ranking second only to lung cancer by 2030 [3].

Within pancreatic cancer, over 90% is pancreatic ductal adenocarcinoma (PAAD), which has been evident to be insensitive to neither chemotherapy nor radiotherapy, and the emerging immunotherapy is also showing little efficiency in PAAD [4, 5]. Therefore, curative surgery is still considered the main option for PAAD treatment. However, attributing to the indistinctive symptoms during the early stage of disease, distant metastasis has occurred to 50 ~ 60% patients at the time they were hospitalized [1, 6]. And even if curative surgeries were performed at early stage, the recurrence and subsequent metastasis rate is still as high as 60% within the first 12 months after surgery [7]. The high mortality makes it urgent to explore clearly of the cancer genetic events thus developing novel and more effective molecular targeted therapies, identifying promising prognostic indicators and potential drug targets, improving the survival of patients suffering from pancreatic cancer.

Over the past few decades, multiple genes have been reported to play threatening roles in PAAD development, including four main driver genes, namely KRAS, CDKN2A, TP53 and SMAD4 [8, 9]. Within the four genes, KRAS is the most frequently mutated oncogene which occurs in over 90% PAAD, resulting in the continuous activation of multiple downstream signaling pathways, for instance, RAS-RAF-MAPK signaling and PI3K-AKT signaling pathway [8]. As opposed to KRAS, CDKN2A is a tumor suppressor gene relating with cell cycle regulation, however, the inactivation of the gene also occurs in over 90% PAAD, resulting from various mechanisms, including homozygous deletion (40%), heterozygous deletion (40%) and promoter methylation (10 ~ 15%) [8]. Meanwhile, the somatic mutation of TP53 gene happens in 75% and the inactivation of SMAD4 in 55% PAAD patients, the former has been a well known tumor suppressor gene and the latter plays an important role in the regulation of TGF-beta signaling pathway [8].

Besides the above genes, various studies also discovered new potential driver genes in PAAD development, for instance, KDM6A, PREX2 and RREB1, which mutations were reported to occur in 10 ~ 18% PAAD patients [10, 11]. Moreover, some genetic susceptibility genes which have been proven to burden on breast and ovarian cancer, including BRCA1, BRCA2, PALB2 and PTEN were also evident to be carried in PAAD patients^12^, ^13^.

However, despite above rising understanding of the genetic events in PAAD development, the clinical molecular targeting therapy is woefully lacking comparing to the highly heterogeneous, complicated and progressive cancer nature. Besides the recent breakthrough in KRAS targeting drugs that a newly developed AMG510 has been showing great response in KRAS G12C positive lung and colorectal cancer [14–16], indicating its future potential use in pancreatic cancer, only numbered molecular drugs including olaparib and PARP inhibitors were approved by FDA for PAAD patients with BRCA mutation [17–20]. Limited improvement were received over the decades for molecular targeting therapy in PAAD patients, making it vital to keep digging and understanding the genetic information of PAAD, thus identifying promising survival indicators and new potential drug target-able genes.

In the modern precise medicine era, the emerging high throughput molecular pathological detection technologies, for example protein microarray, digital PCR and next generation sequencing (NGS)have been bringing in tremendous diseases data, making it more convenient for worldwide researchers to identify promising disease-causing gene alterations and better understand the genetic basis of cancer development.

In the study, multiple public PAAD datasets and bioinformatic analysis were used to explore the disease date for identifying potential responsible genes. Firstly, five different GEO PAAD cDNA expression profiles GSE15471, GSE16515, GSE41368, GSE43795 and GSE71989 containing a total of 98 cancer and 71 normal pancreatic samples were used to identify the differently expressed genes in PAAD versus normal pancreatic tissues, followed by measuring the difference level. And after the basic genes interpretation by GO and KEGG of the main cellular location, biological function and signaling pathways that the differently expressed genes were enriched in, Kaplan–Meier survival and Cox regression analysis assisted us for identifying two genes namely IMUP and GPRC5A that independently indicate patients both overall and recurrence free survival. Further, more detailed information about IMUP and GPRC5A including their physicochemical properties, their association with immune cells infiltration, the mutation ratio, copy number variation and methylation ratio in pancreatic cancer were analyzed. Last but not least, the expression discrepancy of IMUP and GPRC5A in PAAD comparing to normal tissues were validated by immunohistochemistry(IHC) experiment using local hospital patients samples and the clinical pathological significance were analyzed using TCGA data. The results shall provide promising insights for unearthing potential new prognostic indicators and drug targets for further PAAD clinical treatment.

## Materials and methods

### Data source: cDNA expression profiles from GEO database

Five cDNA expression profiles GSE15471 [21], GSE16515 [22], GSE41368 [23], GSE43795 [24] and GSE71989 [25] were selected from GEO database for exploring the differently expressed genes in PAAD comparing to normal pancreatic tissues. The GEO profiles selection criteria were set as: 1. profiles data were based on human tissues; 2. covering both PAAD cancer and normal pancreatic samples results; 3. containing at least 10 samples.

Of the five selected profiles, GSE15471 was based on GPL570 platform [HG-U133_Plus_2] Affymetrix Human Genome U133 Plus 2.0 Array, containing 36 PAAD and 36 normal pancreatic samples. GSE16515 was based on GPL570 platform [HG-U133_Plus_2] Affymetrix Human Genome U133 Plus 2.0 Array, containing 36 PAAD and 16 normal pancreatic samples. GSE71989 was based on GPL570 platform [HG-U133_Plus_2] Affymetrix Human Genome U133 Plus 2.0 Array, containing 13 PAAD and 8 normal tissues. GSE43795 was based on GPL10558 platform Illumina Human HT-12 V4.0 expression beadchip, containing 7 PAAD and 5 normal samples. And GSE41368 was based on GPL6224 platform [HuGene-1_0-st] Affymetrix Human Gene 1.0 ST Array [transcript (gene) version] and contains 6 PAAD and 6 normal samples (Detailed in Table S1).

### Datasets processing: identify differently expressed genes in PAAD vs normal pancreatic tissues

GEO2R [26] has been a widely used gene expression analysis service which is commonly provided paring with GEO profiles online. In the study, GEO2R was used to screen the differently expressed genes in PAAD comparing to normal pancreatic samples with the criteria set as adjusted P value < 0.05. The candidate genes were then classified into 4 groups according to |log2FC| value as: |log2FC|< 1, |log2FC|≥ 1, |log2FC|≥ 2 and |log2FC|≥ 3, namely the genes’ expression discrepancy level was < twofold, 2 ~ fourfold, 4 ~ eightfold and > eightfold in each group. We mainly focused on the > eightfold gene cluster for further GO and KEGG genes interpretation and subsequent analysis.

### GO and KEGG interpretation of the high level differently expressed genes in PAAD

Gene ontology analysis (GO) and Kyoto Encyclopedia of Genes and Genomes (KEGG) [27] have been effectively used to interpret the characteristic biological attributes of multiple genes, including the main biological processes, molecular functions and the signaling pathways they mainly enriched in. In the study, we separately yet simultaneously analyzed the biological functions of the < twofold, 2 ~ fourfold, 4 ~ eightfold and > eightfold group of genes. Considering the feasibility of further clinical pathology validation using immunohistochemistry experiment, the greater difference level genes were higher regarded, especially the over eightfold gene cluster.

### Risk score assessment of the high level gene cluster

SurvExpress [28] is a newly developed cancer-wide gene expression database with clinical outcomes and a web-based tool that provides survival analysis and risk assessment, containing more 20,000 samples covering over 20 cancer types, thus facilitating the validation of multiple candidate genes for survival risk assessment. In the study, after understanding the basic biological features of different gene groups using GO and KEGG, the eightfold genes were further analyzed and those who were shared in multiple GEO profiles were especially selected as a candidate gene cluster, followed by nest step SurvExpress risk score assessment.

### Kaplan–Meier survival and Cox regression analysis identify the key genes regulating PAAD development

Kaplan–Meier plotter (KM) [29] has been a widely used open access online service for overall survival (OS) and recurrence free survival (RFS) analysis which contains over 10,000 samples, allowing the assessment of the association between 54,000 genes and 21 different types cancers survival. In the study, we used KM analysis to orderly access the effect on PAAD survival of above selected candidate gene cluster, the genes that were indicated to statistical significantly correlate with PAAD survival by KM would then be processed for next step multivariate COX regression analysis. The genes that were supported by both KM analysis and Cox regression for associating with PAAD survival would be identified as potential key genes during PAAD development and processed for further detailed interpretation.

### Physicochemical properties analysis of key genes

ProtParam [30] and ProtScale [31] are two effectively used online service for computing the physical and chemical parameters of selected proteins including their theoretical isoelectric point, molecular weight, aminoacid composition, estimated protein half life, protein instability index, hydrophobicity, hydrophilicity and secondary structure conformational parameters.

In addition, GeneCards [32] has also been widely used for interpreting the basic information of certain genes, which is an openly accessed human genes centered knowledgebase, providing comprehensive information including genomic, transcriptomic, proteomic and clinical data on basically all annotated and predicted genes.

Meanwhile, Human Protein Atlas [33] is developed based on multiple useful molecular biological technologies for instance antibody-based imaging, mass spectrometry-based proteomics, transcriptomics and systems biology for mapping various certain proteins in cells, tissues and human organs.

In the study, GeneCards was used to understand the basic genetic information of selected key genes, and Human Protein Atlas was performed to explore the genes cellular location and basic expression situation of key genes in PAAD, followed by ProtParam and ProtScale analyzing the genes’ physicochemical properties.

### GEPIA expression validation of key genes

Gene Expression Profiling Interactive Analysis (GEPIA) [34] has been an effectively used web-based service which is constructed based on TCGA and GTEx programs data for worldwide researchers to perform certain gene’s differential expression analysis, profiling plotting, correlation analysis, similar gene detection and dimensionality reduction analysis, facilitating deeper data mining and more precise understanding of certain gene functions. In the study, GEPIA was used to preliminary validate the expression change of selected key genes in PAAD comparing to normal pancreatic tissues.

### Key genes’ association with immune cells infiltration

TIMER [35] is a comprehensive web resource for systematical evaluating the infiltration of multiple types of immune cells including CD4 + T cell, CD8 + T cell, B cell, neutrophil, macrophage, monocyte, NK cell and cancer associated fibroblast in diverse cancer types. The web is based on 10,009 samples across 23 cancer types from TCGA, being effectively used for analyzing the survival correlation of given immune cells in certain cancer types or evaluating the association between the expression of a certain gene and diverse immune cell types. In the study, TIMER2.0 was used to explore the association between the expression of selected key gene with immune cells infiltration in PAAD.

### Genetic alteration analysis of key genes in PAAD

Besides the mRNA expression difference, other types of variations of the selected key genes including mutation ratio, copy number variation, amplification and deletion ratio, methylation and phosphorylation in PAAD were explored based on cBioPortal database [36], aiding more precise understanding of the potential regulation of key genes on PAAD development.

cBioPortal is one of the largest open access cancer genomics data website, which integrates over 126 large-scale tumor research projects and covers more than 2,8000 cancer samples. After logging into the cBioPortal website, the “cancer types summary” module of “quick search” section was the mostly used part for querying the genetic alteration characteristics of previous selected key genes in various cancer types, of which PAAD was specially focused. Meanwhile, the “mutation” module was also used to display the mutated site information of key genes in 3D protein structures.

### PAAD tissue microarray production

The PAAD patients samples used for tissue microarray production were all from local hospital surgeries and stored at our Pathology Department Biobank after routine pathological examination. Informed consent of the potential scientific application of surgery tissues have been obtained from patients at the same time the samples were stored in the Biobank, and the use of the sample in the study was approved by the Hospital Institutional Board (Second Hospital of ShanXi Medical University, China).

Originally, 100 PAAD patients samples with complete clinical information were picked from Biobank, after pathological confirmation of pure PAAD diagnosis without mixing with other types of pancreatic cancer and evaluation of the cancer percentage, 62 samples were proceeded for further microarray production. Two independent areas were circled in each sample to eliminate tumor heterogeneity and further being planted in receptor wax block using 1.5 mm needle according to manual operating instructions (Chloe, BeiJin, China). Then, sample slides were obtained by serial sectioning of the receptor wax block and stored at 4 °C refrigerator preparing for further use.

### Immunohistochemistry (IHC) experiment

#### Regents and tissue samples

IHC experiment was conducted using PAAD tissue microarray to validate the different expression of selected key genes in cancer comparing to normal pancreatic tissues. The experiment was performed on VENTANA platform (Roche) using local hospital Pathology Department equipment and regents. The primary antibody of key genes: anti-GPRC5A and anti-IMUP were both purchased from abcam (NO.ab155557 and ab221063 respectively). The secondary antibody (Envision /HRP kit) and DAB detection kit were from ZSBG-Bio purchased by Pathology Department, and other reagents including H2O2, phosphate-buffered saline (PBS), antigen retrieval citrate solution and hematoxylin stain were all from hospital Supply Department.

#### IHC experimental protocol

The 4 °C stored PAAD tissue microarray slides were rewarmed at room temperature for 30 min before they were used for IHC experiment according to the operating procedures as described before. In brief, the slides were deparaffinized, rehydrated, treated with 0.3% H2O2 for inhibiting endogenous peroxidase activity, boiled in 10 mmol/l citrate buffer for antigen retrieval and incubated with specific primary and secondary antibodies, followed by being visualized in DAB for final results evaluation by pathologists.

#### IHC results evaluation

IHC experiment result was evaluated by two experienced pathologists registered in our hospital Pathology Department (Second Hospital of ShanXi Medical University, China) according to both the staining intensity and staining area. Cellular membrane and cytoplasm staining was regarded as positive for GPRC5A gene. And any part of cancer cell including membrane, cytoplasm or nuclear staining could be considered positive for IMUP gene. The criteria for staining intensity evaluation was same for both GPRC5A and IMUP genes, being set as: none (0), mild (1), moderate (2) and strong (3), at the same time, the staining area was classified as: < 5% (0), 6–25% (1), 26–50% (2), 51–75% (3) and > 75% (4), the final score equals the multiplication of staining intensity and staining area. The cut-off was set as 4, if the final score < 4, the result would be recorded as negative, and if score ≥ 4, it would be classified as positive.

### Association between key genes expression and PAAD clinical pathological parameters

Ualcan [37] is a comprehensive and user-friendly integrated data-mining platform for analyzing cancer transcriptome data. In the study, Ualcan was effectively used to analyze the association between key genes’ expression and PAAD clinical parameters.

Moreover, besides Ualcan, the clinical information of 182 TCGA PAAD samples (Detailed in Table S2) which were previously used for Cox regression analysis were also applied for exploring key genes’ clinical significance.

## Results

### GEO data identified 22,491 differently expressed genes in PAAD vs. normal pancreatic samples

**Fig. 1:**
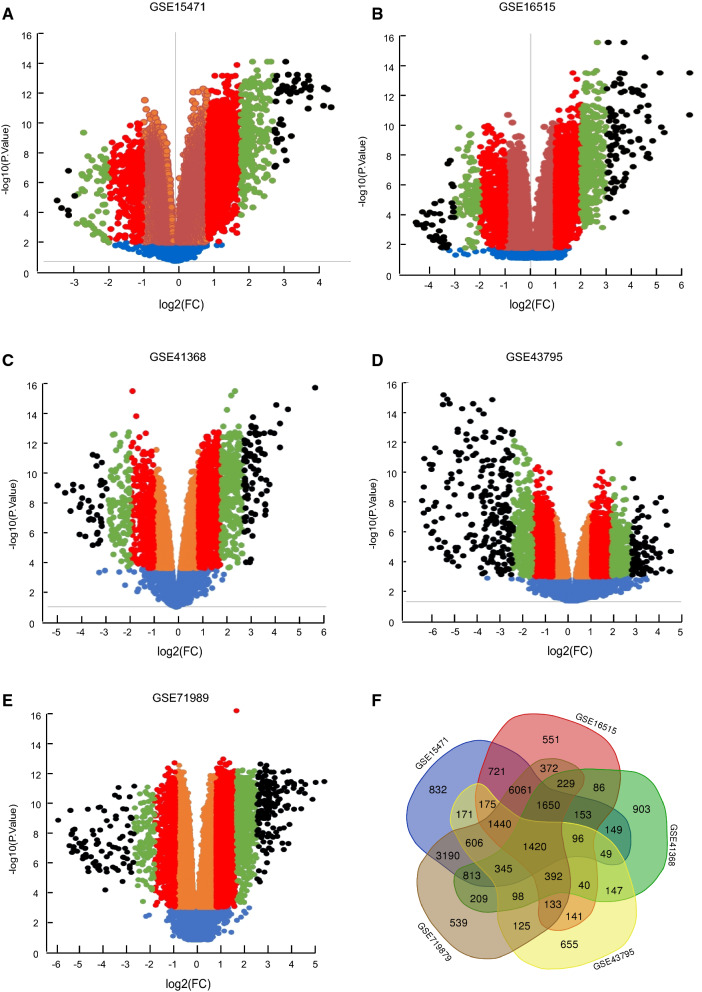
The differently expressed genes in PAAD vs normal pancreatic tissues identified from multiple GEO datasets. From GEO datasets **A** GSE15471, **B** GSE16515, **C** GSE413687, **D** GSE43795 and **E** GSE71989, the up-regulated (right-sided) and down-regulated (left sided) differently expressed genes in PAAD comparing to normal pancreatic tissues were identified. And the genes were then classified into four groups based on difference level as: < twofold genes (orange-colored spots in **A**–**E**), 2 ~ fourfold genes (red-colored spots in A-E), 4 ~ eightfold genes (green-colored spots in **A**–**E**) and > eightfold genes (black-colored spots in **A**–**E**). **F** The intersection of the genes in different GEO profiles

Five different GEO cDNA expression profiles were combine used to explore the differently expressed genes between PAAD and normal pancreatic tissue, and 17871, 13660, 6779, 6003 and 17622 genes were identified in GSE15471 (Fig. 1A), GSE16515 (Fig. 1B), GSE41368 (Fig. 1C), GSE43795 (Fig. 1D) and GSE71989 (Fig. 1E) respectively. Besides the genes that were shared in different profiles, a total of 22,491 genes were eventually identified (Fig. 1F).

To understand the genes’ expression more precisely, the candidate genes were then classified into 4 groups based on the discrepancy level. To avoid the data deviation caused by different GEO platforms, the five profile genes were analyzed separately. And the result showed that in GSE15471, the expression change of 13511 genes were less than twofold, 1503 genes were 2 ~ fourfold, 211 genes were 4 ~ eightfold and 38 genes were over eightfold in cancer vs normal pancreatic tissues (Fig. 1A). In GSE16515, the gene number was 9379, 1414, 601 and 187 in each group respectively (Fig. 1B). In GSE41368, the gene number was 4350, 1110, 358 and 98 in each group respectively (Fig. 1C). In GSE43795, the gene number was 3291, 1996, 524 and 275 respectively (Fig. 1D). Meanwhile, in GSE71989, the gene number was 11631, 3084, 601 and 187 in < twofold, two ~ fourfold, four ~ eightfold and > eightfold group respectively (Fig. 1E).

### High level expression changing genes were mainly located in extracellular space

**Fig. 2:**
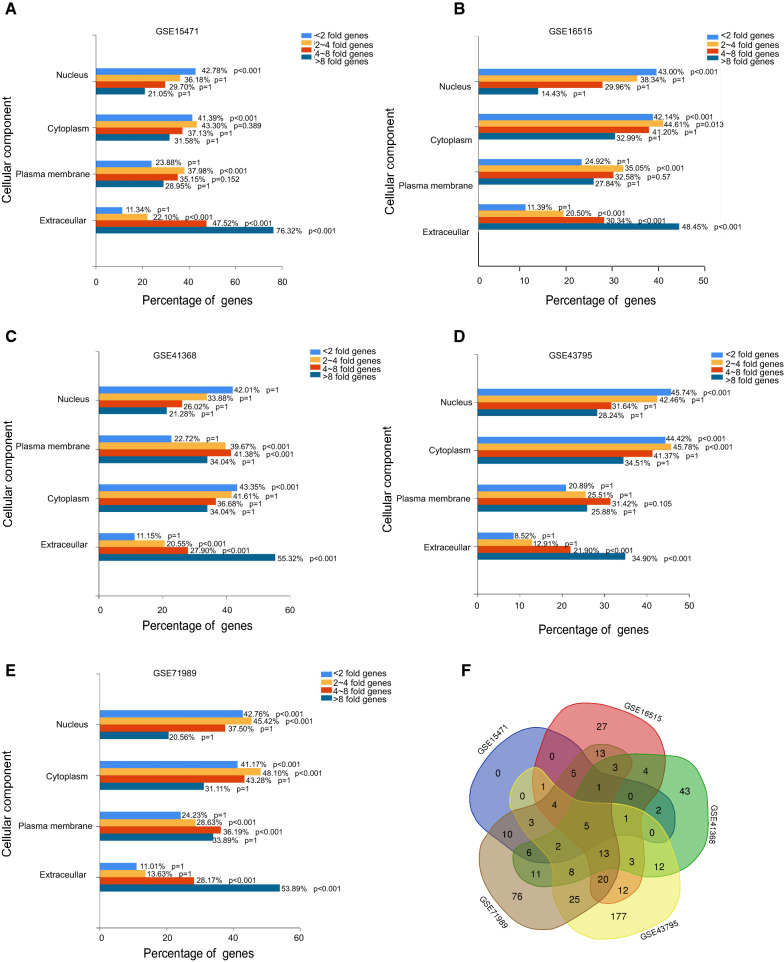
Cellular location interpretation of the differently expressed in PAAD. The mainly enriched cellular components of the four groups (< twofold genes as the light blue bar, two ~ fourfold as the yellow bar, four ~ eightfold as the orange bar and > eightfold genes as the dark blue bar) of differently expressed genes in **A** GSE15471, **B** GSE16515, **C** GSE413687, **D** GSE43795 and **E** GSE71989 respectively. **F** The intersection of the > eightfold genes that were mainly located in the extracellular region revealed by multiple GEO profiles

To explore the potential biological functions of each group of differently expressed genes, GO and KEGG analysis were performed. Interestingly, the result of all five profiles revealed an inspiring fact that the < twofold genes were mainly located in the nuclear, and four ~ eightfold genes were mostly in the cytoplasm and > eightfold genes were tend to locate in extracellular region (Fig. 2A–E), indicating an interesting trend that the more different genes expression are, their cellular location tend to become more outwards from the cell nuclear, which is consist with the biology common sense that most function genes are synthesised in nuclear and regulated by various modular factors, the slight change in nuclear protein might result in massive extra nuclear proteins changes.

Meanwhile, for the convenience of further immunohistochemistry (IHC) experiment validation and potential clinical use, we mainly focused on the high discrepancy eightfold genes for further analysis since they are more potentially to be popularized for clinical test.

**Fig. 3:**
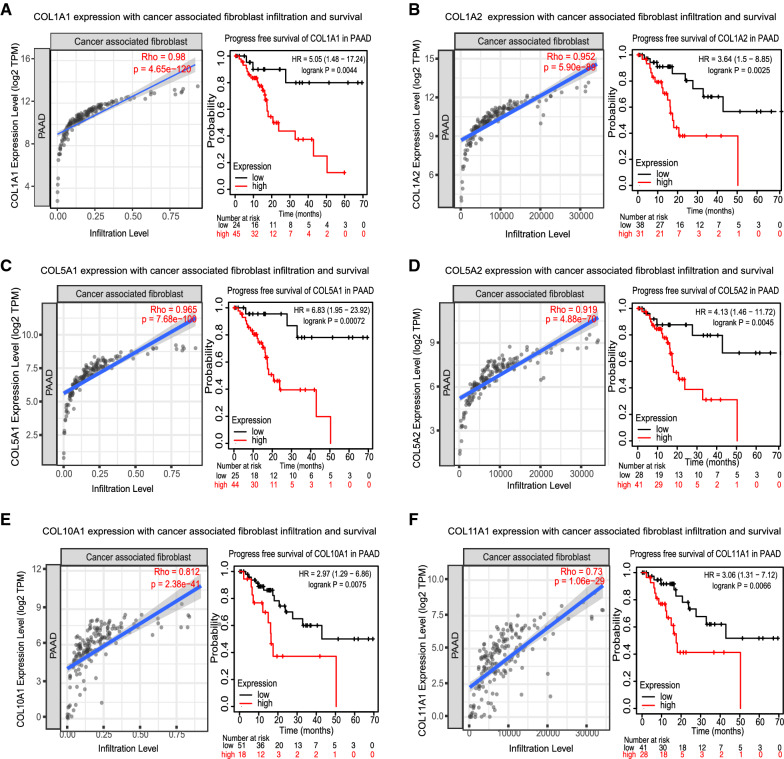
The association between collagen related genes and PAAD survival. The association between collagen related regulators **A** COL1A1, **B** COL1A2, **C** COL5A1, **D** COL5A2, **E** COL10A1, **F** COL11A1 and PAAD cancer associated fibroblasts infiltration (Left graphic) and PAAD patients recurrence free survival (right graphic)

Further interpretation of the 487 over eightfold genes showed that a total of 55/487 genes were shared in at least 3/5 profiles indicating their expressions were more convinced to be different in cancer comparing to normal tissues (Fig. 2F), and a large percent of these genes were collagen related regulators, for instance COL1A1, COL1A2, COL5A1, COL5A2, COL10A1 and COL11A1, which were associated with PAAD cancer related fibroblasts infiltration and directly affect patients recurrence free survival (Fig. 3A–F).

### Risk score assessment of high level genes

**Fig. 4:**
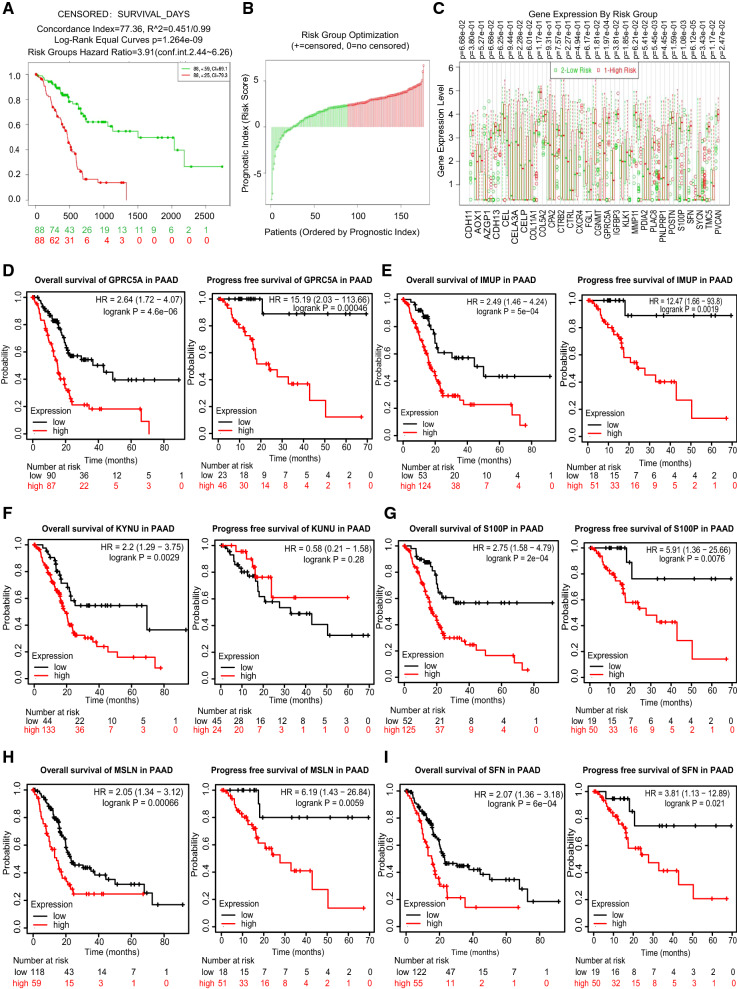
Risk score assessment and KM survival to identify the candidate key genes. **A** The Risk score assessment of the > eightfold extracellular locating genes which result separates the PAAD patients into two groups with high and low survival risk respectively. **B** Risk score assessment displayed according to the prognostic index of each patient (green bars as the low rick group of patients, and red as the patients with high risk index). **C** The relative expression of the 55 > eightfold extracellular locating genes in the high and low risk patients groups. The overall survival (left graphic) and recurrence free survival (right graphic) analysis of **D** GPRC5A, **E** IMUP, **F** KYNU, **G** S100P, **H** MSLN and **I** SFN in PAAD

After preliminary understanding the basic information of eightfold genes, SurvExpress Risk assessment of the 55 genes supported their potential prognostic indicating functions in PAAD development, with the concordance index equals 77.36, risk group hazard ratio equals 3.91, the 55 genes expression by risk group were also explored (Fig. 4A–C).

### Survival analysis identified GPRC5A and IMUP as two independent prognostic indicators in PAAD

To further scale down the candidate genes, KM survival which is based on GEO, EGA and TCGA data and Ualcan survival which is based on TCGA, MET500 and CPTAC data were combine used to analyze the overall survival of the 55 genes. The result showed that 6/55 genes were revealed by both KM and Ualcan to be associated with PAAD survival, namely GPRC5A (Fig. 4D), IMUP (Fig. 4E), KYNU (Fig. 4F), MSLN (Fig. 4G), S100P (Fig. 4H) and SFN (Fig. 4I).

Further, multivariate cox regression analysis showed that tumor Grade, GPRC5A expression and IMUP gene expression were independent prognostic indicators in PAAD development (Table 1).

**Table 1:** Multivariate cox regression analysis on PAAD overall survival

Variables	Pancreatic ductal adenocarcinoma		
	Hazard ratio	95% CI	P value
Grade	1.802	1.072 ~ 3.029	0.026
GPRC5A expression < median vs > median	3.002	1.224 ~ 1.361	0.016
IMUP expression < median vs > median	5.411	1.928 ~ 15.183	0.001

### Physicochemical properties of GPRC5A and IMUP genes

ProtParam, ProtScale and Protein Atlas analysis were successively used to interpret the physiochemical information of GPRC5A and IMUP. The results revealed that GPRCGA protein is composed of 357 amino acids with an estimated molecular weight as 40.3KD, and the protein half-life time is computed to be 30 h in mammals. The amino acids composition of GPRC5A includes 29 negatively charged amino acid residues (ASP + Glu) and 32 positively charged amino acid residues (Arg + Lys), and the protein theoretical isoelectric point is computed to be 8.39.

**Fig. 5:**
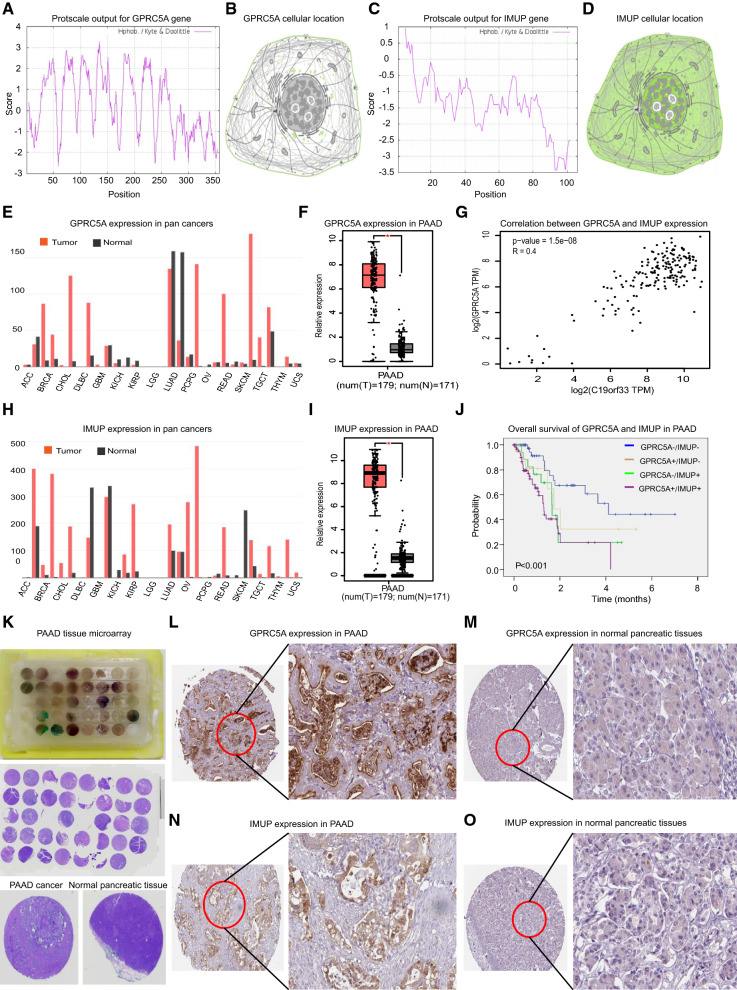
Aberrant GPRC5A and IMUP gain of expression in PAAD comparing to normal pancreatic tissues. **A** The hydrophilcity / hydrophobicity analysis of GPRC5A protein by ProtScale. **B** The prediction model of the cellular location of GPRC5A protein. **C** The hydrophilcity / hydrophobicity analysis of IMUP protein by ProtScale. **D** The prediction model of the cellular location of IMUP protein. **E** Expression of GPRC5A in different types of human cancers. **F** Aberrant gain of expression of GPRC5A in PAAD comparing to normal pancreatic tissues. **G** The correlation analysis of GPRC5A and IMUP in PAAD.** H** Expression of IMUP in different types of human cancers. **I** Aberrant gain of expression of IMUP in PAAD comparing to normal pancreatic tissues. **J** Overall survival analysis of four group of PAAD patients with GPRC5A-/IMUP-, GPRC5A-/IMUP + , GPRC5A + /IMUP- and GPRC5A + /IMUP + expression respectively. **K** One of the tissue arrays made by local hospital PAAD samples and the HE staining of the array. The relative expression of GPRC5A in **L** PAAD cancer and **M** normal pancreatic tissues revealed by IHC experiment of the tissue array. The relative expression of IMUP in **N** PAAD cancer and **O** normal pancreatic tissues revealed by IHC experiment of the tissue array

Meanwhile, the estimated instability index of GPRC5A is 38.49 indicating it’s a cellular stable protein. The ProtParam computed grand average of hydrophobic value is 0.310 which is consistent with the ProtScale analysis result which showed GPRC5A protein harbors more hydrophobicity regions than hydrophilic regions, indicating it’s a hydrophobic protein (Fig. 5A). Moreover, the result of Protein Atlas analysis supported GPRC5A locating in plasma membrane and cellular vesicles (Fig. 5B).

As for IMUP protein, ProtParam revealed the protein is composed of 106 amino acids, containing 13 negatively charged amino acid residues (ASP + Glu) and 21 positively charged amino acid residues (Arg + Lys) with the estimated protein theoretical isoelectric point as 9.73. The molecular weight of IMUP protein is 10.9KD, and the half-life time in mammals is 30 h with an instability index computed to be 38.42 indicating it’s also a cellular stable protein.

Additionally, the ProtParam estimated grand average of hydrophobic value of IMUP protein is -1.372, and ProtScale also revealed IMUP protein harbors multiple hydrophilic regions and shall be classified as a hydrophilic protein (Fig. 5C). And Protein Atlas supported IMUP probably locates in nucleoplasm, cytosol and plasma membrane (Fig. 5D), indicating its potential biological function as a hydrophilic protein involving in various signaling pathways.

### Aberrant GPRC5A and IMUP gain of expression in PAAD

GEPIA was used to validate the change of expression of GPRC5A and IMUP genes in PAAD comparing to normal pancreatic tissues, and the result revealed that although GPRC5A gene expression various in different human tumors (Fig. 5E), for instance, its expression was lower in kidney clear cell renal cell carcinoma and lung squamous cell carcinoma comparing to the matched normal tissues, its expression in most other types of tumors including PAAD (Fig. 5F) was aberrant significantly higher than matched normal tissues.As for IMUP gene, a honest similar result was observed which showed that although the expression various in multiple human tumors (Fig. 5H), the expression was significantly higher in PAAD comparing to normal pancreatic tissues (Fig. 5I).

Interestingly, pearson correlation analysis indicated a positive connection between the two genes, indicating their potential regulation of similar signaling pathways during PAAD development (Fig. 5G). And the survival analysis showed that the patients with both GRPC5A and IMUP positive harbor the worst survival, next the patients with either GPRC5A or IMUP positive, and the survival of patients with both genes negative was the best (Fig. 5J).

Besides the online data analysis, the results of IHC as well as qRT-PCR experiments which were performed on local hospital patients samples also validated the aberrant gain of expression of GPRC5A and IMUP in PAAD. The IHC experiment conducted on PAAD tissue microarray verified that the positive ratio of both GPRC5A and IMUP were much higher in cancer (74.6 and 46.8% respectively) than matched normal pancreatic samples (both less than 5%), supporting the aberrant gain of expression of GPRC5A and IMUP in PAAD (Fig. 5K–O).

### Other types of genetic alteration analysis

**Fig. 6:**
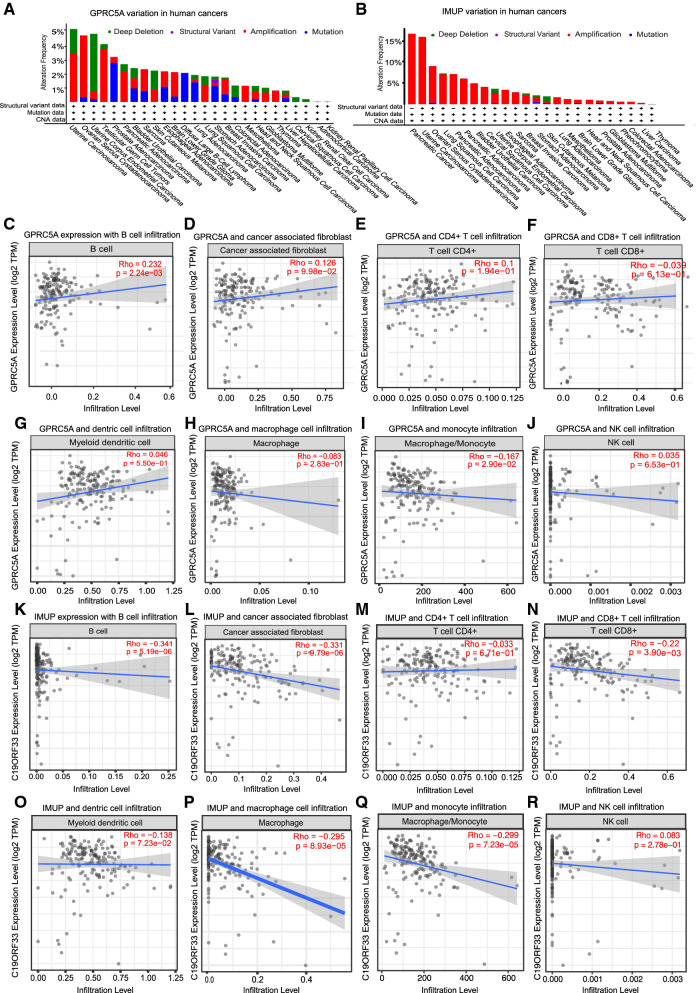
GPRC5A and IMUP genes variations and the association with immune cells infiltration in PAAD. Different types of **A** GPRC5A and **B** IMUP variations including deletion, structural variate, amplification and mutation in various human cancers revealed by cBioPortal dataset. The relation between GPRC5A gene expression and **C** B cell, **D** cancer associated fibroblast, **E** CD4 + T cell, **F** CD8 + T cell, **G** dentric cell, **H** macrophage cell, **I** monocyte and **J** NK cell infiltration in PAAD. The relation between IMUP gene expression and **K** B cell, **L** cancer associated fibroblast, **M** CD4 + T cell, **N** CD8 + T cell, **O** dentric cell, **P** macrophage cell, **Q** monocyte and **R** NK cell infiltration in PAAD

Besides mRNA expression, other GPRC5A and IUMP alterations including mutation ratio, protein structure variant and copy number variation were preliminary explored based on cBioPortal database. The results revealed that as for GPRC5A gene, the gene variation type differs in various tumors, a certain percent of gene mutation, deletion and amplification occurs in multiple human tumors. But in PAAD, gene amplification was the main type of alteration, which ratio was significantly higher than deletion (Fig. 6A). Meanwhile, although several mutation sites in GPRC5A gene were reported in human tumors, they were mainly discovered in other tumors, for instance uterine endometrioid carcinoma, lung squamous cell carcinoma and bladder urothelial carcinoma, none mutation has been tested for GPRC5A gene in PAAD.

As for IMUP gene, the gene variation types were much less than GPRC5A gene, the amplification was the main type of gene variation in multiple human tumors, only numbered of deletion or mutation was discovered, and none of them were reported in PAAD (Fig. 6B).

The patients with GPRC5A and IMUP genes altered (all types of alteration besides amplification) showed worse overall, disease free and progression free survival than patients without gene alterations, indicating the genes’ potential value in cancer development (data not shown).

### Association between GPRC5A and IMUP gene expression and PAAD immune cells infiltration

Immune cells infiltration in cancer has been a well known important component of tumor microenvironment, which not only relating with the ability of cancer initiation, progression and metastasis, but also associating with the effect of immune targeting therapy. To evaluate the potential association between GPRC5A and IMUP genes expression with PAAD immune cell infiltration, TIMER database was used. However, the result revealed that none significant correlation was found between GPRC5A gene expression and CD4 + T cell, CD8 + T cell, B cell, NK cell, monocyte, macrophage cell, dendritic cell or cancer associated fibroblast infiltration in PADD (Fig. 6C–J). Meanwhile, none prominent association was found between IMUP expression and above immune cells infiltration in PAAD neither (Fig. 6K–L).

### Clinical significance of GPRC5A and IMUP genes in PAAD development

**Fig. 7:**
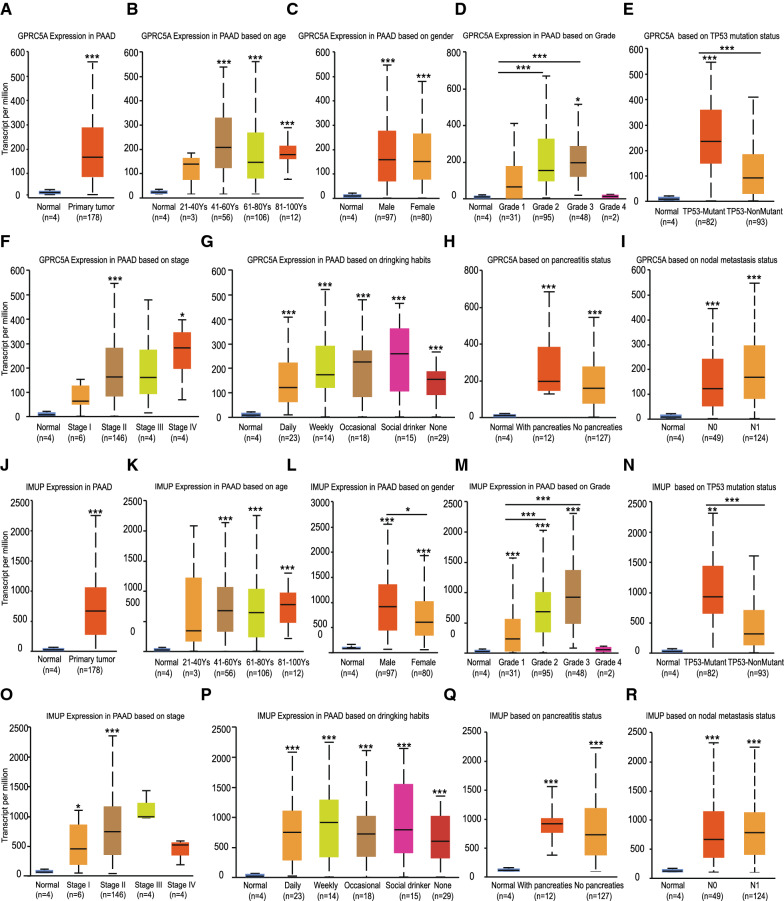
The association between GPRC5A, IMUP expression and PAAD clinical parameters. **A** Relative GPRC5A expression in PAAD versus normal pancreatic tissues. And the association between GPRC5A expression and PAAD **B** patients age, **C** gender, **D** cancer grade, **E** TP53 gene mutation, **F** cancer stage, **G** patients drinking habit, **H** chronic pancreatitis history and **I** lymph node metastasis. **J** Relative IMUP expression in PAAD versus normal pancreatic tissues. And the association between IMUP expression and PAAD **K** patients age, **L** gender, **M** cancer grade, **N** TP53 gene mutation, **O** cancer stage, **P** patients drinking habit, **Q** chronic pancreatitis history and **R** lymph node metastasis. (* p < 0.05, **p < 0.01, ***p < 0.001. The first layer * which is right above the error bar representing comparison to normal group, and the above layers * which were above a secondary line represent the comparison between corresponding groups that were covered by the line)

To access the association between GPRC5A and IMUP expression with PAAD clinical parameters, we used two methods. Firstly, an online Ualcan service was used, and the result showed that not only the GPRC5A and IMUP genes expression were markedly higher in cancer comparing to normal samples (Fig. 7A, J), but also the expression of both genes keep increasing as the cancer stage and grade advancing (Fig. 7D, F, M, O). Also, as for GPRC5A gene, it tends to express more in patients with node metastasis (Fig. 7I) and heavy drinking habit (Fig. 7G), and IMUP gene favors to express in male (Fig. 7L) and elder patients (Fig. 7K), but the difference was not statistical significant. Meanwhile, no significance relationship was found between GPRC5A and IMUP expression with patients pancreatitis status (Fig. 7H, Q). Interestingly, a worth emphasizing interaction was found between GPRC5A and IMUP genes expression with P53 mutation that both genes expression were significantly higher in patients with P53 mutation than the patients without P53 variation indicating the potential association between GPRC5A and IMUP genes with TP53 related signaling pathways (Fig. 7E, N).

Part of above online analyzing results were further validated by another analysis, during which we downloaded the original clinical information of 182 PAAD samples from TCGA website (the same data used for previous multivariate Cox Regression analysis), and the results verified the trend that both GPRC5A and IMUP genes expression increasing as the cancer grade advancing. However, although the genes expression were higher in more advanced stage patients, the difference were not statistical significant potentially because the limited patients number in stage III and IV group. Meanwhile, none specific association was found between genes expression and other elements for instance patients race, age, gender or distant metastasis, partly attributing to the limited samples in some groups (Tables 2, 3).

**Table 2:** The association between GPRC5A and PAAD clinical pathological features

Parameters	GPRC5A(%)		P Value
	−	+	
Gender			
Male	32 (42.5%)	43 (57.5%)	0.579
Female	50 (46.7%)	57 (53.3%)	
Race			
White	93 (59.2%)	64 (40.8%)	0.315
Black	5 (83.3%)	1 (16.7%)	
Asia	5 (45.5%)	6 (54.5%)	
Subdivision			
Head of Pancreas	82 (59.4%)	56 (40.6%)	0.285
Body of Pancreas	7 (50.0%)	7 (50.0%)	
Tail of Pancreas	7 (46.7%)	8 (53.3%)	
Age			
< 60 years	33 (55.2%)	27 (44.8%)	0.472
≥ 60 years	74 (60.8%)	48 (39.2%)	
Alcohol habit			
No	58 (62.3%)	29 (37.7%)	0.428
Yes	57 (56.4%)	44 (43.6%)	
Grade			
Grade 1	2 (100.0%)	0 (0.00%)	0.031
Grade 2	24 (77.4%)	7 (22.6%)	
Grade 3	55 (57.9%)	40 (42.1%)	
Grade 4	22 (45.8%)	26 (54.2%)	
Stage			
Stage I	17 (81.0%)	4 (19.0%)	0.101
Stage II	82 (56.8%)	63 (43.2%)	
Stage III	2 (66.7%)	1 (33.3%)	
Stage IV	1 (20.0%)	4 (80.0%)	
T			
T1	7 (77.8%)	2 (22.2%)	0.122
T2	19 (79.2%)	5 (20.8%)	
T3	77 (54.2%)	65 (45.8%)	
T4	2 (66.7%)	1 (33.3%)	
N			
N0	34 (61.8%)	21 (38.2%)	0.608
N1	71 (57.7%)	52 (42.3%)	
M			
M0	44 (55.2%)	35 (44.8%)	0.121
M1	1 (20.0%)	4 (80.0%)

**Table 3:** The association between IMUP and PAAD clinical pathological features

Parameters	IMUP(%)		P Value
	−	+	
Gender			
Male	46 (45.9%)	54 (54.1%)	0.228
Female	45 (55.0%)	37 (45.0%)	
Race			
White	79 (50.3%)	78 (40.7%)	0.670
Black	3 (50.0%)	3 (50.0%)	
Asia	4 (36.4%)	7 (63.6%)	
Subdivision			
Head of Pancreas	68 (49.3%)	70 (50.7%)	0.448
Body of Pancreas	6 (42.9%)	8 (57.1%)	
Tail of Pancreas	7 (46.7%)	8 (53.3%)	
Age			
< 60 years	31 (51.7%)	29 (48.3%)	0.749
≥ 60 years	60 (49.2%)	62 (50.8%)	
Alcohol habit			
No	43 (55.8%)	34 (44.2%)	0.173
Yes	46 (45.5%)	55 (54.5%)	
Grade			
Grade 1	0 (0.0%)	2 (100.0%)	0.004
Grade 2	24 (77.4%)	7 (22.6%)	
Grade 3	43 (45.3%)	52 (54.7%)	
Grade 4	20 (41.7%)	28 (58.3%)	
Stage			
Stage I	15 (71.4%)	6 (28.6%)	0.271
Stage II	69 (47.3%)	77 (52.7%)	
Stage III	1 (33.3%)	2 (66.7%)	
Stage IV	2 (40.0%)	3 (60.0%)	
T			
T1	6 (66.7%)	3 (33.3%)	0.351
T2	15 (62.5%)	9 (37.5%)	
T3	67 (47.2%)	75 (52.8%)	
T4	1 (33.3%)	2 (66.7%)	
N			
N0	28 (50.9%)	27 (49.1%)	0.568
N1	61 (49.6%)	62 (50.4%)	
M			
M0	42 (53.2%)	37 (46.8%)	0.749
M1	2 (40.0%)	3 (60.0%)	

## Discussion

Pancreatic cancer has been a lethal malignancy worldwide and over 90% is pancreatic ductal adenocarcinoma (PAAD). The overall 5-year survival rate was 7 ~ 9% and 1-year rate less than 20%, to make it worse, the cancer is insensitive to neither chemotherapy nor radiotherapy, even the emerging immunotherapy which has been showing promising clinical effect in other tumors receives limited response in pancreatic cancer, making it urgent to explore the potential gene targets and develop drug-targeting therapies. In the study, multiple GEO profiles data and bioinformatic analysis tools were combine used to explore the genetic information of PAAD and select potential responsible genes during cancer development.

Based on five different GEO cDNA expression profiles GSE15471, GSE16515, GSE41368, GSE43795 and GSE71989 which contain a total of 98 PAAD and 71 normal pancreatic samples, we identified 22491 genes that were differently expressed in cancer vs. normal tissues and then classified them into 4 different groups according to the difference level considering the potential unique functions of each group. Interestingly, further interpretation of the 4 groups of genes indicated that the greater the genes expression difference are, their cellular location were more tend to be far away from cell nuclear. More specifically, the expression difference < twofold genes were mainly located in nuclear, and four ~ eightfold genes were mostly in cytoplasm, meanwhile the > eightfold genes were tend to locate on the cell membrane or in extracellular region. The trend makes reasonable sense considering the fact that except for the certain percent of genes that were synthesized in cell mitochondrion, most human proteins were produced in nuclear abiding by the biology “central dogma” that the direction of genetic information flow is from DNA-RNA–protein, the slight change in the nuclear protein might result in massive proteins change extracellular.

Given the convenience of further IHC experiment validation, which is the most common method for clinical medical diagnosis, and the genes shall harbor more chance to be translated into clinical use if they are suitable to be tested by IHC, we mainly focused on the > eightfold genes. Interestingly, detailed analysis of the > eightfold group of extracellular genes revealed that they were mostly extracellular matrix structural constituent regulating related genes, for instance, a certain percent of them were collagen related regulators, which were proven to be associating with PAAD cancer related fibroblasts infiltration, although none direct relation were found between these genes expression and patients overall survival, they certainly affect patients recurrent free survival, stressing the importance of microenvironment construction in cancer development, which is certainly an inspiring direction for further research.

Actually, to identify the potential “unique key genes” during PAAD development, we then combine used KM survival, UALCAN survival and multivariate Cox Regression analysis to successively explore the association between 55 over eightfold genes and PAAD patient survival, and the results highlighted two genes: GPRC5A and IMUP, which were supported by all three analysis to be associated with both patients overall and recurrence free survival and worked as independent prognostic indicators in PAAD development.

GPRC5A, which is short for G Protein-Coupled Receptor Class C Group 5 Member A, is a member of the GPCR family, locating in 12p13.1 and encoding a protein that is characterized by the signature 7-transmembrane domain motif. And based on the computed physicochemical parameters of the protein, GPRC5A is a hydrophobic protein weighting 40.3KD and mainly locating in cellular membrane and extracellular space, the estimated half-time is 30 h and tend to be stable in human cells. Actually, the gene has been reported to play critical roles in embryonic development and epithelial cell differentiation, the dysregulation of GPRC5A was known to be involved in multiple cancers including lung, breast, colon and other types of cancers [5, 38]. In the study, we mainly focused on its potential regulation on PAAD development.

Meanwhile, IMUP, which is more commonly known as C19orf33 being short for chromosome 19 open reading frame 33 and locating on 19q13.2, encodes a hydrophilic protein weighing 10.9KD. And the encoded protein probably locates in nucleoplasm, cytosol and plasma membrane, with the estimated half-time as 30 h in human cells and potentially relates with human placental development. The aberrant dysregulation of IMUP has been reported in endometrial carcinoma and pre-eclampsia [39–41].

Interestingly, besides the strong correlation between GPRC5A and IMUP genes expression indicating their potentially similar biological functions and close involving signaling pathways in PAAD, the aberrant higher expression of both genes were indicated to be relating with worse patients both overall survival and recurrence free survival, supported not only by previous online analysis, but also IHC experiments using local hospital PAAD tissue array which was produced using 62 local PAAD patients samples and matched normal pancreatic tissues. A more inspiring discovery is that based on the expression of these two genes, pancreatic cancer patients could be divided into 4 groups, the patients with neither GRPC5A nor IMUP expression shows much better prognosis than the patients with either gene expression, and the survival of patients with both genes expression was the worst in 4 groups, indicating the drug developing potentiality of the two genes.

Besides the mRNA expression, other types of variation of GPRC5A and IMUP were also explored which results revealed basically a similar result that the gene amplification works as the main variation type for both GPRC5A and IMUP in PAAD, only occasional gene mutation or deletion occurs. Moreover, none specific relation was found between neither gene expression nor immune cells infiltration.

Additionally, to evaluate the potential correlation between GPRC5A and IMUP expression with PAAD clinical parameters, both UALCAN online analysis and TCGA original clinical data were used, both results indicated the genes were more higher expressed in patients with more advance cancer stage and grade, supporting the potential clinical value of the genes for indicating cancer developing. Recent reports highlighted that GPRC5A and IMUP were involved in several human cancers via participated in various tumor-associated signaling pathways, for instance the nuclear factor (NF)-κB [42–44], signal transducer and activator of transcription (STAT) 3 [45, 46], and focal adhesion kinase (FAK)/Src signaling [47–51], however, they are still on the way to be validated in PAAD.

Although the current result is not yet enough to classify GPRC5A or IMUP as new useful clinical drug targets, comprehensive studies and clinical trials are needed to confirm the findings before promoting the clinical utility of the genes in PAAD clinical treatment. Meanwhile, further detailed correlation of the two genes, as well as their regulation on PAAD biological processes including cancer proliferation, invasion, migration and apoptosis are urgently need to be performed. The results shall provide meaningful insight into better understanding of the disease. We sincerely hope the study will provoke worldwide researchers’ interest to further explore the pancreatic cancer deeply and benefit the suffering patients in the near future.

## Conclusion

In conclusion, based on multiple GEO database and bioinformatic analysis tools, we identified 22491 genes that were differently expressed in PAAD comparing to normal pancreatic tissues, and highlighted 2 genes of them: GPRC5A and IMUP as independent prognostic indicators in cancer development. Both online public data analysis and local hospital IHC experiment validated the aberrant up regulation of genes in PAAD. Kaplan–Meier, UALCAN survival and cox regression analysis supported that high GPRC5A and IMUP genes expression were associated with worse patients survival. Basic physiochemical properties, other types variations and association with immune cells infiltration were preliminary explored. Above results shall provide meaningful insights into better understanding of the molecular mechanism behind PAAD development, comprehensive studies and biological experiments are needed to confirm the findings before promoting the clinical utility of the genes in PAAD clinical.

## Supplementary Information

**Additional file 1: Table S1**. Detailed GEO datasets information used for identifying the candidate differently expressed genesin PAAD vs normal pancreatic tissues. **Table S2**. The TCGA patients barcodefor182 PAAD samples.

## Data Availability

All data generated or analyzed during this study are included in this published article.

## Abbreviations

PAAD: Pancreatic ductal adenocarcinoma
GEO: Gene Expression Omnibus
GO: Gene ontology
KEGG: Kyoto Encyclopedia of Gene and Genome
IHC: Immunochemistry experiment
qRT-PCR: Quantitative real time polymerase chain reaction
OS: Overall survival rate
RFS: Progress free survival rate
GPRC5A: G Protein-Coupled Receptor Class C Group 5 Member A
IMUP: Chromosome 19 open reading frame 33
GEPIA: Gene Expression Profiling Interactive Analysis
