# Supplementary material for: IMUP and GPRC5A: two newly identified risk score indicators in pancreatic ductal adenocarcinoma
Source: Cancer Cell Int. 2021 Nov 24;21:620. doi: 10.1186/s12935-021-02324-w (PMC8613923; doi:10.1186/s12935-021-02324-w)
Supplement: Supplementary file 1 — Additional file 1: Table S1. Detailed GEO datasets information used for identifying the candidate differently expressed genesin PAAD vs normal pancreatic tissues. Table S2. The TCGA patients barcodefor182 PAAD samples. [file 12935_2021_2324_MOESM1_ESM.docx]

#### Supplementary Table 1 Detailed GEO datasets information used for identifying the candidate differently expressed genes in PAAD vs normal pancreatic tissues

| **GEO Dataset** | **Public data** | **Contributor** | **Sample amount** | **Accessing website** |
| --- | --- | --- | --- | --- |
| GSE15471 | Jun 13, 2009 | Liviu Badea | 36 PAAD and  36 normal N | https://www.ncbi.nlm.nih.gov/geo/query/acc.cgi?acc=GSE15471 |
| GSE16515 | Jun 10, 2009 | Liewei Wang | 36 PAAD and  16 normal N | https://www.ncbi.nlm.nih.gov/geo/query/acc.cgi?acc=GSE16515 |
| GSE41368 | Dec 30, 2013 | Teresa Colombo | 6 PAAD and  6 normal N | https://www.ncbi.nlm.nih.gov/geo/query/acc.cgi?acc=GSE41368 |
| GSE43795 | Dec 31, 2013 | minhee park | 7 PAAD and  5 normal N | https://www.ncbi.nlm.nih.gov/geo/query/acc.cgi?acc=GSE43795 |
| GSE71989 | Aug 13, 2015 | Thomas Schmittgen | 13 PAAD and  8 normal N | https://www.ncbi.nlm.nih.gov/geo/query/acc.cgi?acc=GSE71989 |

#### Supplementary Table 2 The TCGA patients barcode for 182 PAAD samples

| TCGA-2J-AAB1 | TCGA-2J-AABF | TCGA-2J-AABU | TCGA-3A-A9I7 | TCGA-3A-A9I5 | TCGA-3A-A9IX |
| --- | --- | --- | --- | --- | --- |
| TCGA-2J-AAB4 | TCGA-2J-AABH | TCGA-2J-AABV | TCGA-3A-A9IB | TCGA-3A-A9IZ | TCGA-HV-AA8V |
| TCGA-2J-AAB6 | TCGA-2J-AABI | TCGA-2L-AAQA | TCGA-3A-A9IC | TCGA-3A-A9J0 | TCGA-HV-AA8X |
| TCGA-2J-AAB8 | TCGA-2J-AABK | TCGA-2L-AAQE | TCGA-3A-A9IH | TCGA-3E-AAAY | TCGA-HZ-7289 |
| TCGA-2J-AABA | TCGA-2J-AABO | TCGA-2L-AAQI | TCGA-3A-A9IJ | TCGA-3E-AAAZ | TCGA-HZ-7918 |
| TCGA-2J-AABE | TCGA-2J-AABP | TCGA-2L-AAQJ | TCGA-3A-A9IL | TCGA-F2-6879 | TCGA-HZ-7919 |
| TCGA-2J-AAB9 | TCGA-2J-AABR | TCGA-2L-AAQL | TCGA-3A-A9IN | TCGA-F2-6880 | TCGA-HZ-7920 |
| TCGA-3A-A9I9 | TCGA-2J-AABT | TCGA-2L-AAQM | TCGA-3A-A9IO | TCGA-F2-7273 | TCGA-HZ-7922 |
| TCGA-F2-A8YN | TCGA-FB-AAQ2 | TCGA-HZ-7923 | TCGA-3A-A9IS | TCGA-F2-A44G | TCGA-HZ-8315 |
| TCGA-FB-A4P5 | TCGA-FB-AAQ3 | TCGA-HZ-7924 | TCGA-3A-A9IU | TCGA-F2-A44H | TCGA-HZ-8317 |
| TCGA-FB-A4P6 | TCGA-FB-AAQ6 | TCGA-HZ-7925 | TCGA-3A-A9IV | TCGA-F2-A7TX | TCGA-HZ-8519 |
| TCGA-FB-A545 | TCGA-H6-8124 | TCGA-HZ-7926 | TCGA-3A-A9IR | TCGA-F2-7276 | TCGA-HZ-8636 |
| TCGA-FB-A5VM | TCGA-H6-8124 | TCGA-HZ-8001 | TCGA-HZ-A77P | TCGA-IB-8127 | TCGA-HZ-8637 |
| TCGA-FB-A78T | TCGA-H6-A45N | TCGA-HZ-8002 | TCGA-HZ-A77Q | TCGA-IB-A5SO | TCGA-HZ-8638 |
| TCGA-FB-A7DR | TCGA-H6-A45N | TCGA-HZ-8003 | TCGA-HZ-A8P0 | TCGA-IB-A5SP | TCGA-HZ-A49G |
| TCGA-FB-AAPP | TCGA-H8-A6C1 | TCGA-HZ-8005 | TCGA-RB-AA9M | TCGA-IB-A5SQ | TCGA-HZ-A49H |
| TCGA-FB-AAPQ | TCGA-HV-A5A3 | TCGA-HZ-A8P1 | TCGA-RL-AAAS | TCGA-IB-A5SS | TCGA-HZ-A49I |
| TCGA-FB-AAPS | TCGA-HV-A5A3 | TCGA-IB-AAUW | TCGA-S4-A8RM | TCGA-IB-A5ST | TCGA-HZ-A4BH |
| TCGA-FB-AAPU | TCGA-HV-A5A4 | TCGA-IB-AAUP | TCGA-S4-A8RO | TCGA-IB-A6UF | TCGA-HZ-A4BK |
| TCGA-FB-AAPY | TCGA-HV-A5A5 | TCGA-IB-7645 | TCGA-S4-A8RP | TCGA-IB-A6UG | TCGA-HZ-A77O |
| TCGA-FB-AAPZ | TCGA-HV-A5A6 | TCGA-US-A77G | TCGA-US-A774 | TCGA-IB-A7LX | TCGA-US-A77J |
| TCGA-FB-AAQ0 | TCGA-HV-A7OL | TCGA-OE-A75W | TCGA-IB-7890 | TCGA-IB-A7M4 | TCGA-XD-AAUG |
| TCGA-FB-AAQ1 | TCGA-HV-A7OP | TCGA-PZ-A5RE | TCGA-IB-7891 | TCGA-IB-7886 | TCGA-XD-AAUH |
| TCGA-HZ-A9TJ | TCGA-IB-AAUM | TCGA-Q3-A5QY | TCGA-IB-7893 | TCGA-IB-7887 | TCGA-XD-AAUI |
| TCGA-HZ-A9TJ | TCGA-IB-AAUN | TCGA-Q3-AA2A | TCGA-IB-7897 | TCGA-IB-7888 | TCGA-XD-AAUL |
| TCGA-IB-7644 | TCGA-IB-AAUO | TCGA-RB-A7B8 | TCGA-IB-8126 | TCGA-IB-7889 | TCGA-XN-A8T3 |
| TCGA-IB-7646 | TCGA-IB-AAUQ | TCGA-L1-A7W4 | TCGA-US-A776 | TCGA-YB-A89D | TCGA-XN-A8T5 |
| TCGA-IB-7649 | TCGA-IB-AAUR | TCGA-LB-A7SX | TCGA-US-A779 | TCGA-YH-A8SY | TCGA-YB-A89D |
| TCGA-IB-7651 | TCGA-IB-AAUS | TCGA-LB-A8F3 | TCGA-US-A77E | TCGA-YY-A8LH | TCGA-IB-AAUV |
| TCGA-IB-7652 | TCGA-IB-AAUT | TCGA-LB-A9Q5 | TCGA-M8-A5N4 | TCGA-Z5-AAPL | TCGA-IB-7885 |
| TCGA-IB-7654 | TCGA-IB-AAUU |  |  |  |  |
